# Cross species application of quantitative neuropathology assays developed for clinical Alzheimer’s disease samples

**DOI:** 10.1080/20010001.2019.1657768

**Published:** 2019-09-03

**Authors:** Silvan R. Urfer, Caitlin S. Latimer, Warren Ladiges, C. Dirk Keene, Sarah Benbow, Benjamin Harrison, Daniel E.L. Promislow, Matt Kaeberlein, Brian C Kraemer, Adrienne Wang, Franco Guscetti, Martin Darvas

**Affiliations:** a Department of Pathology, University of Washington, Seattle, WA, USA; b Department of Comparative Medicine, University of Washington, Seattle, WA, USA; c Department of Medicine, University of Washington, Seattle, WA, USA; d Department of Psychiatry and Behavioral Sciences, University of Washington, Seattle, WA, USA; e Veterans Affairs Geriatric Research Education and Clinical Center, Seattle, WA, USA; f Department of Biology, Western Washington University, Bellingham, WA, USA; g Institute of Veterinary Pathology, Vetsuisse Faculty, University of Zürich, Zürich, Switzerland

**Keywords:** Luminex, amyloid β42, phospho-Tau, Alzheimer’s disease, cortex

## Abstract

A major obstacle for preclinical testing of Alzheimer’s disease (AD) therapies is the availability of translationally relevant AD models. Critical for the validation of such models is the application of the same approaches and techniques used for the neuropathological characterization of AD. Deposition of amyloid-β 42 (Aβ42) plaques and neurofibrillary tangles containing phospho-Tau (pTau) are the pathognomonic features of AD. In the neuropathologic evaluation of AD, immunohistochemistry (IHC) is the current standard method for detection of Aβ42 and pTau. Although IHC is indispensable for determining the distribution of AD pathology, it is of rather limited use for assessment of the quantity of AD pathology. We have recently developed Luminex-based assays for the quantitative assessment of Aβ42 and pTau in AD brains. These assays are based on the same antibodies that are used for the IHC-based diagnosis of AD neuropathologic change. Here we report the application and extension of such quantitative AD neuropathology assays to commonly used genetically engineered AD models and to animals that develop AD neuropathologic change as they age naturally. We believe that identifying AD models that have Aβ42 or pTau levels comparable to those observed in AD will greatly improve the ability to develop AD therapies.

**Abbreviations**: Alzheimer’s disease (AD); amyloid β 42 (Aβ42); phospho-Tau (pTau); immunohistochemistry (IHC)

Alzheimer’s disease (AD) is the most common cause of dementia in the US, accounting for 60–80% of all cases in 2016 [1]. The hallmark neuropathological features of AD are accumulation of amyloid-β 42 (Aβ42) in plaques and neurites, and of phospho-Tau (pTau) in neurofibrillary tangles and dystrophic neurites, both of which are the basis for the current National Institute on Aging-Alzheimer’s Association (NIA-AA) guidelines for the neuropathological assessment of AD [2]. Hence, any model of AD must have Aβ42 and/or pTau pathology and should ideally be validated for the neuropathological features of AD using procedures that mirror those in human AD [3]. However, the NIA-AA diagnostic criteria are not quantitative measures, which limits their utility in studies that investigate the relationship between AD pathology with parameters such as sex, genotype, age, or environmental factors. Objective and quantitative measures of AD pathology are also essential for the testing of AD interventions. Recently, a Luminex-based approach was developed to quantify Aβ42 and pTau in frozen and formalin-fixed brain tissue from AD patients [4].

In an effort to investigate whether this precisely quantitative and highly sensitive assay can also be used for the validation of animal models used in aging and AD research, we collected tissue from various animal species and subjected them to the same technical and analytical procedures as were previously used for human AD tissue. We used genetic mouse (*Mus musculus*), fly (*Drosophila melanogaster*) and nematode (*Caenorhabditis elegans*) models that are commonly used in AD research; they are listed with detailed information in Table 1 [5–11]. Because age is the greatest risk factor for AD, we also analyzed two animal species with reported findings of age-associated AD neuropathologic changes that occur naturally: aged (21 years) Caribbean vervets (*Chlorocebus aethiops sabaeus*), and aged (12 years) dogs (*Canis lupus familiaris*, Maltese and miniature Schnauzer). The vervets were born and raised in the Vervet Research Colony (P40-OD010965, PI Jay R. Kaplan) and housed at Wake Forest School of Medicine where all procedures involving monkeys were conducted in accordance with state and federal laws, standards of the Department of Health and Human Services, and guidelines established by the Wake Forest Institutional Animal Care and Use Committee. Vervet samples were obtained from Dr. Suzanne Craft (Wake Forest Alzheimer’s Disease Center). Dog samples from privately owned aged dogs that had died of natural non-neurological cases and did not have a clinical history of cognitive decline were retrieved from the archives of the Institute for Veterinary Pathology, Vetsuisse Faculty, University of Zürich, Zürich, Switzerland. All mouse tissue samples were collected from experiments that were approved by the Institutional Animal Care and Use Committee at the University of Washington. All dog-brain samples were formalin fixed and all other samples were frozen and without any fixatives.

**Table 1.** Genetic strains of mutant animals used in this study.

Strain/Name	Species	Mutations	Pathology
APPswe/PSEN1dE9	*Mus musculus*	APP (KM670/671NL), PSEN1 (deletion of exon 9)	Aβ42 expression in multiple brain regions
CL2659	*Caenorhabditis elegans*	Aβ42 minigene	Aβ42 expression in muscle tissue
GMR-GAL4> UAS-Aβ; Tau	*Drosophila melanogaster*	Aβ42 minigene, wild-type *MAPT* gene	Aβ42 and wild-type Tau expression in eye
PS19	*Mus musculus*	MAPT P301S	Phosphorylated Tau in multiple brain regions
CK10	*Caenorhabditis elegans*	MAPT V337M	Phosphorylated Tau in neurons
CK144	*Caenorhabditis elegans*	Wild-type *MAPT* gene	Wild-type tau expression in neurons

For mice, vervets and dogs, we extracted proteins from cortical regions that correspond to the human frontal cortex. For the invertebrate species, we extracted proteins from complete fly heads and whole-worm pellets. The same extraction procedures that we used for human brain samples were used for all animal samples to generate RIPA-buffer (contain pTau) and guanidine-hydrochloride (Gu-HCl, contain Aβ42) soluble fractions [4]. Total protein content was determined for each fraction using BCA assays (Pierce, Rockford IL). All samples were analyzed in triplicates and reagent blanks with phosphate-buffered saline (PBS) were always included to determine the background signal of each assay. Only signals with magnitudes of at least five times the response of PBS were interpreted as positive signals indicating the reliable presence of Aβ42 or pTau.

For quantification of Aβ42, we used the same Aβ42 standards, antibodies and procedures as described for analysis of human AD samples [4]. In short, we generated antibody-coupled (monoclonal antibody clone H31L21, Life Technologies, Carlsbad CA) magnetic Luminex beads for Aβ42-antigen capture and biotinylated antibodies (monoclonal antibody clone 6E10, Bio Legend, San Diego CA) for Aβ42-antigen detection. Samples were analyzed in 96-well plates with 200 ng of Gu-HCl fraction per well. We analyzed three cortex samples from aged (21-months) wild-type (WT) C57Bl/6 mice, three cortex samples from 21-months old APPswe/PSEN1dE9 mice, three cortex samples from dogs, two cortex samples from vervets, 200 mg of WT packed worm pellets distributed into 5 samples, 50 mg of CL2659 packed worm pellets (each pellet contained ~50,000 adult animals) distributed into two samples, and four sets of control (W1118, the non-transgenic background for GMR-GAL4> UAS-Aβ;Tau) and GMR-GAL4> UAS-Aβ;Tau fly heads (10 heads per set). The fluorescence intensity (FI) of the Aβ42 Luminex signal measured in aged dog and vervet cortex samples (Figure 1(a)) was significantly above PBS background FI (ANOVA, F_2, 4_ = 206.2, p < 0.01; post hoc comparisons with PBS were significant for dog and vervet, p < 0.01; vervet samples had significantly higher FIs than dog samples, p < 0.01). Aβ42 FIs were also significantly higher than PBS background in cortical samples from APPswe/PSEN1dE9 mice (Figure 1(b)) but not in WT mice (ANOVA, F_3, 6_ = 561.1, p < 0.01; post hoc comparisons with PBS were only significant for APPswe/PSEN1dE9 mice, p < 0.01). Similarly, only whole-worm preparations from the CL2659 strain (Figure 1(c)) had significantly elevated levels of Aβ42 FIs (ANOVA, F_2, 6_ = 1458, p < 0.01; post hoc comparisons with PBS were only significant for CL2659 worms, p < 0.01), and only fly-head preparations from the GMR-GAL4> UAS-Aβ;Tau strain (Figure 1(d)) had significantly elevated levels of Aβ42 FIs (ANOVA, F_2, 7_ = 40.99, p < 0.01; post hoc comparisons with PBS were only significant for GMR-GAL4> UAS-Aβ;Tau flies, p < 0.01). Importantly, in all samples with significantly elevated Aβ42 FIs, the measured FIs were always more than five times the response of the assay blank (PBS). We then used the FIs measured in Aβ42 standards together with 5-parameter logistic equations to calculate the amount of Aβ42 present in all samples with FIs that were significantly above PBS background (Figure 1(e)). To provide better context for the absolute quantification of Aβ42 in animal samples, we also show previously published values for Aβ42 levels in human frontal cortex of patients with or without AD (dotted lines in Figure 1(e)).

**Figure 1.**
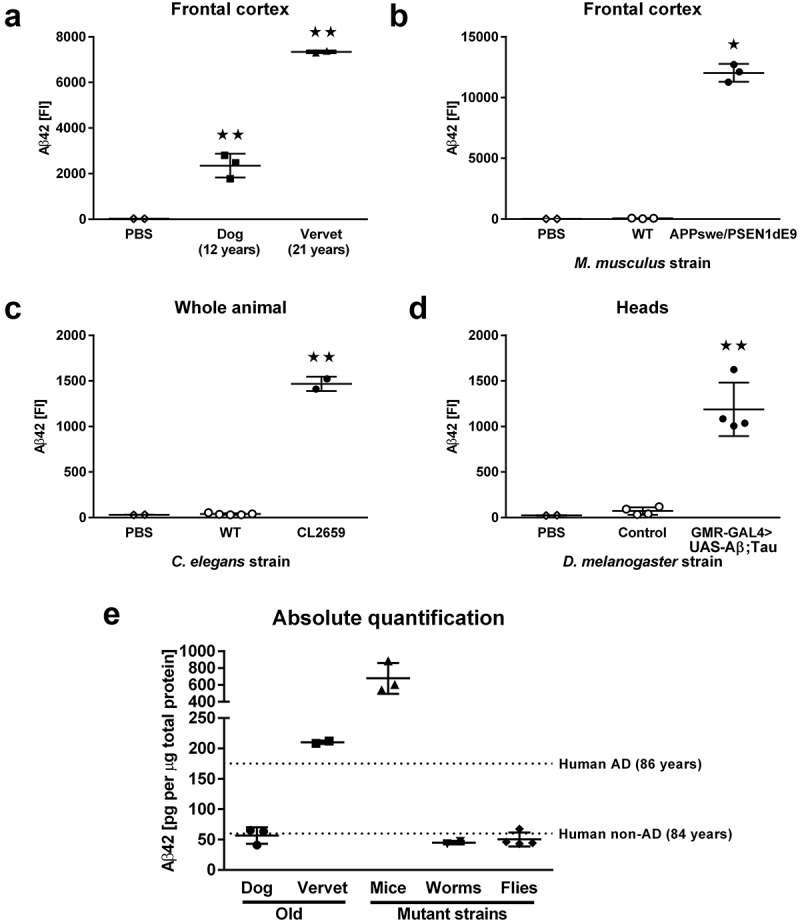
Quantification of Aβ42. (a) Fluorescent-intensity (FI) signals for Aβ42 in guanidine-soluble extracts from frontal cortex in 12-year old dogs (n = 3) and 21-year old vervets (n = 2). (b) FI signals for Aβ42 in guanidine-soluble extracts from frontal cortex in 21-month old WT (n = 3) and APPswe/PSEN1dE9 mice (n = 3). (c) FI signals for Aβ42 in guanidine-soluble extracts from whole-body worm pellets in WT (n = 5) and CL2659 worms (n = 2). (d) FI signals for Aβ42 in guanidine-soluble extracts from fly heads in control (n = 4) and GMR-GAL4> UAS-Aβ;Tau flies (n = 4). (e) Concentrations of Aβ42 in guanidine-soluble extracts with Aβ42 FIs that were significantly elevated above background (PBS) levels; as a reference guide, we show averages from previously published [4] findings in AD frontal cortex samples (dashed lines). Samples were analyzed with ANOVA followed by post hoc pairwise comparisons. All data are presented as mean ± SEM. ★ p < 0.05 and ★★ p < 0.01 for post hoc pairwise comparisons.

For quantification of pTau, we used the same antibodies and procedures as described for analysis of human AD samples [4]. In short, we generated antibody-coupled (monoclonal antibody clone AT8, Life Technologies, Carlsbad CA) magnetic Luminex beads for pTau-antigen capture and biotinylated antibodies (monoclonal antibody clone HT7, Life Technologies, Carlsbad CA) for pTau-antigen detection. Samples were analyzed in 96-well plates with 2000 ng of RIPA fraction per well. We analyzed cortical RIPA-soluble fractions from the same aged dogs and vervets as described for Aβ42. We further analyzed two cortex samples from 9-months old WT C57Bl/6 mice, three cortex samples from 9-months old PS19 mice, and 50 mg of WT, CK10 and CK144 worm pellets distributed into two samples each. The FI of the pTau Luminex signal measured in aged dog and vervet cortex samples (Figure 2(a)) was not significantly above PBS background (significant effect of group by ANOVA, F_2, 4_ = 12.46, p < 0.05; no significant post-hoc differences in comparisons with PBS, p > 0.05). pTau FIs were significantly higher than PBS background in cortical samples from PS19 mice (Figure 2(b)) but not in WT mice (ANOVA, F_2, 4_ = 287.1, p < 0.01; post hoc comparisons with PBS were only significant for PS19 mice, p < 0.01). Although only whole-worm preparations from CK10 and CK144 strains (Figure 2(c)) had significantly elevated levels of pTau FIs (ANOVA, F_3, 4_ = 47.12, p < 0.01; post hoc comparisons with PBS were only significant for CK10 and CK144 strains, p < 0.05), all measured FIs for pTau detection in *C. elegans* strains were less than five times the response of the assay blank (PBS). To provide better context for the quantification of AT8-reactive pTau in animal samples with substantial FIs (i.e. transgenic mice), we also show previously published FIs for AT8-reactive pTau that we detected in human frontal cortex of patients with or without AD (dotted lines in Figure 2(b)).

**Figure 2.**
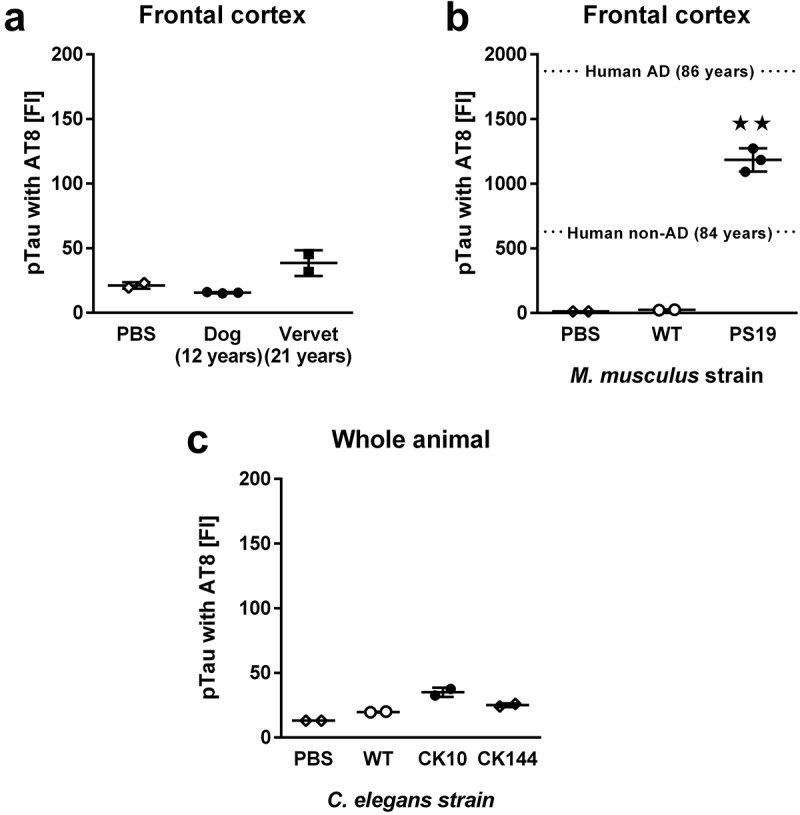
Quantification of pTau using the AT8 antibody. (**a)** FI signals for pTau in RIPA-soluble extracts from frontal cortex in 12-year old dogs (n = 3) and 21-year old vervets (n = 2). (**b)** FI signals for pTau in RIPA-soluble extracts from frontal cortex in 21-month old wild-type (WT, n = 2) and PS19 mice (n = 3). (**c)** FI signals for pTau in RIPA-soluble extracts from whole-body worm pellets in WT, CK10 and CK144 worms (each n = 2). As a reference guide, we show averages from previously published [4] findings in AD frontal cortex samples (dashed lines) in panel **B**. Samples were analyzed with ANOVA followed by post hoc pairwise comparisons. All data are presented as mean ± SEM. ★★ p < 0.01 for post hoc pairwise comparisons.

To determine whether another Tau phosphorylation site implicated in AD could be detected in vervets, dogs and transgenic worm strains CK10 and CK144, we performed additional tests using a different antibody for pTau-antigen capture. Instead of AT8, we now used the AT270 monoclonal antibody (Life Technologies, Carlsbad CA) for coupling with magnetic beads. While the AT8 antibody specifically detects Tau antigens with phosphorylation at amino acids Ser202 and Thr205, the AT270 antibody recognizes Tau antigens with phosphorylation at amino acid Thr181. We analyzed the same samples as described for the analysis with the AT8 antibody: 96-well plates with 2000 ng of RIPA fraction per well and biotinylated HT7 antibodies for pTau-antigen detection. The FI of the AT270 pTau Luminex signal measured in aged dog and vervet cortex samples (Figure 3(a)) was only significantly above PBS background in vervet samples (significant effect of group by ANOVA, F_2, 4_ = 19.11, p < 0.01; significant post-hoc differences in comparisons with PBS only for vervet cortex with FIs more than five times of PBS, p < 0.05). AT270 pTau FIs were significantly higher than PBS background (Figure 3(b)) in all *C. elegans* samples (ANOVA, F_3, 4_ = 1128, p < 0.01; *post-hoc* comparisons with PBS were significant for all worm strains, p < 0.01). However, only FIs from CK10 and CK144 strains were robustly above five times the response of the assay blank (PBS), and samples from the CK10 strain had significantly elevated AT270 pTau FIs when compared to the CK144 strain (p < 0.01). There is no data set available for AT270-reactive pTau FIs in human cortex samples and we therefore cannot provide a clinical context at this time.

**Figure 3.**
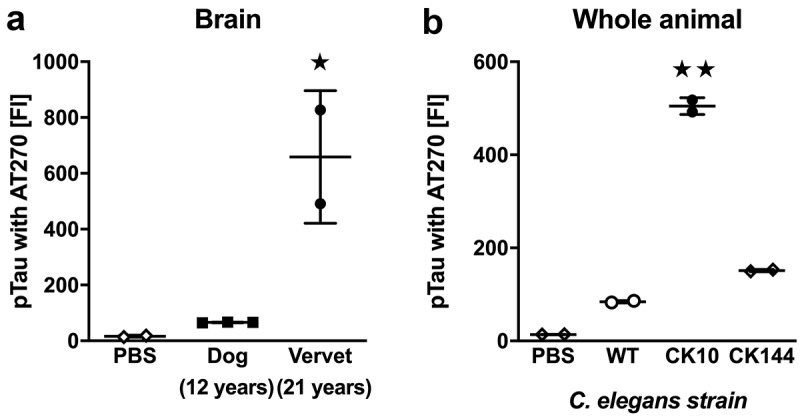
Quantification of pTau using the AT270 antibody. (**a)** FI signals for pTau in RIPA-soluble extracts from frontal cortex in 12-year old dogs (n = 3) and 21-year old vervets (n = 2). (**b)** FI signals for pTau in RIPA-soluble extracts from whole-body worm pellets in WT, CK10 and CK144 worms (each n = 2). Samples were analyzed with ANOVA followed by post hoc pairwise comparisons. All data are presented as mean ± SEM. ★ p < 0.05 and ★★ p < 0.01 for post hoc pairwise comparisons.

These observations suggest that the quantitative Luminex assay that we developed to characterize the amount of Aβ42 in human AD samples can be used without modifications in common genetic laboratory models used in preclinical AD research and also in natural models of aging such as vervets and dogs. The approach for Aβ42 is naturally limited to genetic models or naturally occurring models that share the epitopes of the human Aβ42 sequence that are detected by the H31L21 and 6E10 Aβ antibodies.

The findings in dog brains are of particular interest, because dogs age at roughly seven times the human rate, and as opposed to WT mice develop age-related AD-like pathology spontaneously. They are thus uniquely suited to study AD risk factors and intervention strategies on a much shorter time frame than would be possible in humans or even non-human primates such as vervets. For pTau, our findings indicate that potential inter-species differences of Tau phosphorylation require minor modifications of the pTau Luminex assay that we developed for human AD. While substituting the AT8 pTau capture antibody with the AT270 antibody allowed detection of pTau in aged vervets and mutant *C. elegans* strains, it is possible that additional modifications of the original assay format could still permit Luminex-based quantification of AT8 reactive pTau in those samples. It has been observed that application of NIA-AA guidelines to the analysis of aged vervets identified only ‘not’ and ‘low’ AD neuropathologic change [12]. A comprehensive analysis of pTau in the dog brain by immunohistochemistry found marked hyperphosphorylation of Tau in only three out of 24 study dogs, only one of these three dogs was a small dog comparable to the breeds used in our investigation [13]. Future analyses of larger cohorts that include multiple brain regions are needed to resolve this issue and the question of whether the AT270- or AT8-based Luminex assay format is suitable for the analysis of pTau in aged dogs. Although it is now clear that there Tau becomes hyperphosphorylated in other species, information about the species-specific Tau phosphorylation sites is still incomplete. While some studies were able to detect AT8-reactive pTau or pTau (Ser396) in dog brains [14,15], others could not detect AT8-reactive pTau in dog brains [16,17]. Similarly, some studies report AT8-reactive pTau in aged vervets [12], others detected either no or different pTau epitopes in rhesus monkeys or marmosets [18,19]. It will be of interest to apply our current investigation not only to larger samples, but also to additional species, for example cats [20], that could be used as novel alternative models for the study of AD neuropathologic change.
